# Pbp1-Interacting Protein Mkt1 Regulates Virulence and Sexual Reproduction in *Cryptococcus neoformans*

**DOI:** 10.3389/fcimb.2019.00355

**Published:** 2019-10-17

**Authors:** Ye-Eun Son, Ci Fu, Won-Hee Jung, Sang-Hun Oh, Jin-Hwan Kwak, Maria E. Cardenas, Joseph Heitman, Hee-Soo Park

**Affiliations:** ^1^School of Food Science and Biotechnology, Institute of Agricultural Science and Technology, Kyungpook National University, Daegu, South Korea; ^2^Department of Molecular Genetics and Microbiology, Duke University Medical Center, Durham, NC, United States; ^3^School of Life Science, Handong Global University, Pohang, South Korea

**Keywords:** Mkt1, Pbp1, virulence, *Cryptococcus neoformans*, sexual reproduction

## Abstract

The Mkt1–Pbp1 complex promotes mating-type switching by regulating the translation of *HO* mRNA in *Saccharomyces cerevisiae*. Here, we performed *in vivo* immunoprecipitation assays and mass spectrometry analyses in the human fungal pathogen *Cryptococcus neoformans* to show that Pbp1, a poly(A)-binding protein-binding protein, interacts with Mkt1 containing a PIN like-domain. Association of Pbp1 with Mkt1 was confirmed by co-immunoprecipitation assays. Results of spot dilution growth assays showed that unlike *pbp1* deletion mutant strains, *mkt1* deletion mutant strains were not resistant to heat stress compared with wild-type. However, similar to the *pbp1* deletion mutant strains, the *mkt1* deletion mutants exhibited both, defective dikaryotic hyphal production and reduced pheromone gene (*MF*α1) expression during mating. In addition, deletion of *mkt1* caused attenuated virulence in a murine intranasal inhalation model. Taken together, our findings reveal that Mkt1 plays a crucial role in sexual reproduction and virulence in *C. neoformans*.

## Introduction

*Cryptococcus neoformans* is an opportunistic human pathogenic fungus that causes cryptococcosis, including cryptococcal meningoencephalitis and pulmonary cryptococcosis (Chang et al., 2006; Bratton et al., 2012). *C. neoformans* less commonly causes fungal infection in healthy individuals but does commonly affect those with compromise immunity, including HIV/AIDS patients and organ transplant recipients (Kidd et al., 2004; Park et al., 2009). *Cryptococcus* is the leading cause of adult meningoencephalitis in Sub-Saharan Africa and Southeast Asia and is associated with a high mortality rate (Armstrong-James et al., 2014; Bongomin et al., 2017; Rajasingham et al., 2017). While cryptococcal meningoencephalitis results in a high mortality rate, treatment of cryptococcosis is limited by toxicity of and resistance to current antifungal agents (Perfect et al., 2010; Fisher et al., 2018). Therefore, a comprehensive understanding of biological mechanisms underlying fungal pathogenicity is necessary to develop novel antifungal agents or therapies. Multiple recent studies have conducted comprehensive analyses to obtain insights into the pathogenicity of *C. neoformans* (Liu et al., 2008; Jung et al., 2015; Maier et al., 2015; Gish et al., 2016; Lee et al., 2016).

The Ca^2+^-calmodulin-calcineurin signaling pathway plays a globally conserved role in pathogenicity, stress responses, and host adaptation in pathogenic fungi, including *C. neoformans, Candida albicans*, and *Aspergillus fumigatus* (Bader et al., 2003; Blankenship et al., 2003; Steinbach et al., 2006, 2007a). Loss-of-function mutations in genes encoding components of the calcineurin pathway increase sensitivity of fungi to different environmental stresses and antifungal drugs and attenuate virulence (Steinbach et al., 2007b). Cyclosporine and tacrolimus (FK506) exert antifungal effects on *C. neoformans* by inhibiting calcineurin (Brizuela et al., 1991; Odom et al., 1997). Therefore, elucidation of the molecular mechanisms underlying the calcineurin pathway is important for developing novel antifungal agents (Liu et al., 2015). Calcineurin is a conserved phosphatase activated by the Ca^2+^-calmodulin complex (Rusnak and Mertz, 2000). In *C. neoformans*, activated calcineurin dephosphorylates target proteins to regulate stress responses, cell wall integrity, virulence, and sexual reproduction (Steinbach et al., 2007b). Previously our studies identified 44 calcineurin targets in *C. neoformans* using a phosphoproteomic analysis (Park et al., 2016). Crz1 is a key calcineurin target that regulates mRNA expression of certain target genes (Chow et al., 2017). Along with Crz1, several RNA-binding proteins including Pbp1, Puf4, and Pab1 are potential calcineurin targets in *C. neoformans*.

The poly(A)-binding protein-1 (Pab1)-binding protein (Pbp1) acts downstream of calcineurin to regulate sexual reproduction and virulence of *C. neoformans* (Park et al., 2016; Fu et al., 2018). Results from phosphoproteomic and phosphatase analyses suggest that Pbp1 is a potential calcineurin substrate. A *pbp1*Δ mutant strain showed decreased dikaryotic hyphal production and basidiospore formation, heat resistance, and virulence in a murine inhalation model (Park et al., 2016). Pbp1 colocalizes with calcineurin A in P-bodies and stress granules under thermal stress (Park et al., 2016). In *C. deneoformans* strain XL280α, deletion of *PBP1* conferred high temperature resistance in the presence of FK506 and attenuated virulence (Fu et al., 2018). In *Saccharomyces cerevisiae*, Pbp1 mainly localizes to P-bodies and stress granules under different stress conditions and is involved in RNA metabolism (mRNA polyadenylation and degradation), stress granule assembly, and cell growth (Mangus et al., 1998, 2004a; Swisher and Parker, 2010; Kimura et al., 2013). During stress granule formation, Pbp1 interacts with different proteins, including Pab1, Pbp4, Lsm12, and Mkt1 (Tadauchi et al., 2004; Swisher and Parker, 2010). Although several Pbp1-interacting proteins have been characterized in *S. cerevisiae*, Pbp1-interacting proteins in *C. neoformans* have not been characterized to date.

Mkt1 (Maintenance of K2 Killer Toxin 1) is involved in the maintenance of mitochondrial stability of the K2 killer toxin in *S. cerevisiae* (Wickner, 1980; Dimitrov et al., 2009). Mkt1 forms a complex with Pbp1 (Mkt1–Pbp1 complex) that regulates the translation of *HO* mRNA in *S. cerevisiae* (Tadauchi et al., 2004). Mkt1 localizes to P-bodies in response to environmental stress and maintains mRNA stability by regulating the number of P-bodies (Dimitrov et al., 2009; Lee et al., 2009). In *Trypanosoma brucei*, Mkt1 interacts with Pbp1 and Zc3h11, a zinc finger protein, and plays an important role in post-transcriptional regulatory networks (Singh et al., 2014). However, the role of Mkt1 in *C. neoformans* was unknown. In this study, we identified Mkt1 as a Pbp1-interacting protein and characterized Mkt1 functions in *C. neoformans*. We find that Mkt1 is required for sexual reproduction and virulence of *C. neoformans*.

## Materials and Methods

### Strains and Culture Conditions

Fungal strains used in this study are listed in Table 1. *C. neoformans* strains were grown in liquid or solid yeast extract–peptone–dextrose (YPD) medium (Difco, Sparks, MD, USA) for general culture. To examine heat tolerance, each *C. neoformans* strain was cultured overnight at 30°C in liquid YPD medium. Next, the cultured cells were 10-fold serially diluted, spotted on solid YPD medium, incubated at different temperatures (30, 37, and 39°C), and photographed at 48 or 72 h after treatment.

**Table 1:** *Cryptococcus neoformans* strains used in this study.

**Strains**	**Relevant genotype**	**References**
H99	*MAT*α wild type	Perfect et al., 1980
KN99	*MAT***a** wild type	Nielsen et al., 2003
HP242	*MAT*α *cna1*Δ::*NEO*	Park et al., 2016
HP243	*MAT***a** *cna1*Δ::NEO	Park et al., 2016
HP5	*MAT*α *pbp1*Δ::*NEO*	Park et al., 2016
HP246	*MAT***a** *pbp1*Δ::NEO	Park et al., 2016
HP181	*MAT*α *pbp1*Δ::*NEO PBP1-4xFLAG*::*HYG*	Park et al., 2016
HPC7	*MAT*α *mkt1*Δ::*NEO*	This study
HPC8	*MAT*α *mkt1*Δ::*NEO*	This study
HPC9	*MAT***a** *mkt1*Δ::*NEO*	This study
HPC10	*MAT***a** *mkt1*Δ::*NEO*	This study
HPC11	*MAT*α *mkt1*Δ::*NEO MKT1-4xFLAG*::*HYG*	This study
HPC12	*MAT*α *mkt1*Δ::*NEO MKT1-4xFLAG*::*HYG*	This study
HPC21	*MAT*α *pbp1*Δ::*NEO PBP1-4xFLAG*::*HYG* GFP-*MKT1*::*NAT*	This study
HPC22	*MAT*α *pbp1*Δ::*NEO PBP1-4xFLAG*::*HYG* GFP-*MKT1*::*NAT*	This study
HPC31	*MAT*α *mkt1*Δ::*NEO*	This study
HPC32	*MAT*α *mkt1*Δ::*NEO*	This study

### Generation of *mkt1* Mutant Strains

Oligonucleotides used in this study are listed in Table 2. An *mkt1* deletion allele was generated by transformation with a double-joint PCR, as described previously (Yu et al., 2004). The 5′- and 3′ regions flanking the *MKT1* gene were amplified using primer pairs, JOHE42684–JOHE42686 and JOHE42685–JOHE42687, respectively, and the genomic DNA of *C. neoformans* serotype A H99 strain as the template (Perfect et al., 1993; Janbon et al., 2014). The *NEO* selectable marker was amplified from plasmid pJAF1 (Fraser et al., 2003) with primer pair JOHE40706–JOHE40707. Next, the *mkt1* deletion allele was constructed using primer pair JOHE42688–JOHE42689, the 5′ and 3′ regions flanking *MKT1*, and the *NEO* selectable marker was purified using QIAquick Gel Extraction kit (Qiagen, Valencia, CA, USA). The deletion cassette was combined with gold microcarrier beads (Bio-Rad, Hercules, CA, USA), and the gold bead–DNA particles were introduced into *C. neoformans* strain H99α or KN99**a** via biolistic transformation. Stable transformants were selected on YPD medium supplemented with G418 (Gold Biotechnology, Olivette, MO, USA) and confirmed by diagnostic PCR for two predicted 5′ and 3′ junctions. Multiple deletion mutant strains were obtained by performing independent transformation experiments.

**Table 2:** Oligonucleotide primers used in this study.

**Name**	**Sequence (5^′^ to 3^′^)^a^^,^^b^^,^^c^**	**Purpose**
JOHE40706	GTAAAACGACGGCCAG	*neo* or *nat* marker (M13F)
JOHE40707	CAGGAAACAGCTATGAC	*neo* or *nat* marker (M13R)
JOHE42684	AACTCCCTGGTATCGGTAAGCC	*mkt1* disruption (5F)
JOHE42685	GCACCTAGTGATGATGATGACCC	*mkt1* disruption (3R)
JOHE42686	TCACTGGCCGTCGTTTTAC TGCAGACTGCTGCCTAGTTGAC	*mkt1* disruption with marker (5R)
JOHE42687	CATGGTCATAGCTGTTTCCTG GCGACCATGATTCATGATGTGAC	*mkt1* disruption with marker (3F)
JOHE42688	AGGCAGAATCATCACTGAAGAGG	*mkt1* disruption (NF_Nested)
JOHE42689	GGACGCTGTGGTACAATGGACC	*mkt1* disruption (NR_Nested)
JOHE42724	aatt **GCGGCCGC** GGTAAGCCATGTCGCATCGC	5′*MKT1* with promoter and NotI site
JOHE42725	aatt **GCGGCCGC** TGGTCGCATGGGCTTCAACC	3′*MKT1* with NotI site
JOHE42802	aatt **GGATCC** ATGACTATCCGAGGCTTAGACAG	5′*MKT1* with BamHI site
JOHE42803	aatt **GGATCC** TCATGGTCGCATGGGCTTCA	3′*MKT1* with BamHI site
JOHE42959	CGCCTTCACTGCCATCTTCACC	qPCR_*MF*α*1*_F
JOHE42960	GCGATGACACAAAGGGTCATGC	qPCR_*MF*α*1*_R
JOHE40392	GTCTCTACTGATTTCGTTGGCACTAC	qPCR_*GPD1*_F
JOHE40393	GTAACCGTACTCATTGTCATACCAGCTA	qPCR_*GPD1*_R

a *Underlined sequence is homologous to vector or cassette sequence*.

b *Lowercase sequence indicates linker sequence*.

c *Boldfaced sequence indicates restriction site sequence*.

The *mkt1* + *MKT1* complemented strain was generated by amplifying the *MKT1* ORF and promoter region with primer pair JOHE42724–JOHE42725. PCR products were digested with NotI, purified, and cloned into plasmid pHP1, which contains a 4x FLAG tag (Park et al., 2016). The resulting plasmid pHSP4 was introduced into the recipient *mkt1*Δ (HPC7) strain. Multiple transformants were selected using YPD medium supplemented with hygromycin B (Sigma, St. Louis, MO, USA) and were confirmed by PCR and western blotting.

For co-immunoprecipitation assay, strains expressing GFP-tagged Mkt1 (GFP–Mkt1) and FLAG-tagged Pbp1 (FLAG–Pbp1) were used. For this approach, the *MKT1* ORF was amplified using primer pair JOHE42802–JOHE42803. PCR products obtained were digested with BamHI and cloned into plasmid pCN19 containing a *NAT* marker, a GFP tag and the histone H3 promoter (Price et al., 2008). The resulting plasmid pHSP5 which express GFP-Mkt1 was introduced into the strain HP181 expressing FLAG-Pbp1. Positive transformants were screened using a growth medium supplemented with nourseothricin (Werner BioAgents, Jena, Germany) and were confirmed by performing western blotting. For the control strain, the pHSP5 plasmid was introduced into the recipient HPC7 strain.

### Purification of Pbp1-Interacting Proteins

*Cryptococcus neoformans* strains H99 (WT) and HP181 (*pbp1*Δ + Pbp1–4×FLAG) were grown in liquid YPD medium at 30°C, followed by dilution in fresh YPD medium until the optical density at 600 nm (OD_600_) reached 0.3. The diluted cells were incubated further at 30°C until OD_600_ reached 0.6–0.8, followed by additional incubation at 37°C for 1 h. Next, the cells were collected by centrifugation, washed once with PBS (Sigma), and disrupted in lysis buffer (50 mM Tris-HCl [pH 7.5], 150 NaCl, 0.5 mM EDTA, and 0.5% Triton X-100 containing a protease inhibitor tablet [Roche]) by using 10 cycles of a mini-bead beater (90 s homogenization, with 2 min rest). Cell extracts were centrifuged at 13,000 × *g* for 10 min. Protein concentration of the supernatant was determined using Bradford reagent (Bio-Rad). Next, the supernatant was incubated with anti-FLAG M2 affinity gel (A2220; Sigma), according to the manufacturer's protocol. Immunoprecipitates obtained were centrifuged and washed with PBS containing protease inhibitors. Cells extracts were prepared with cold lysis buffer and cell lysis and immunoprecipitation procedures were carried out at 4°C. Next, the samples were mixed with Laemmli sample buffer and boiled for 5 min. Proteins present in the samples were resolved by SDS-PAGE and visualized by Coomassie blue staining. A stained band from HP181 (*pbp1*Δ + *PBP1*::*4xFLAG*) was excised from the gel and destained. Proteins in the band were digested with trypsin using the In-Gel Tryptic Digestion protocol (abbreviated) available at http://www.genome.duke.edu/cores/proteomics/sample-preparation/.

### Identification of Pbp1-Interacting Proteins

Tryptic peptides were resolved by chromatographic separation with nanoAcquity UPLC (Waters, Milford, MA, USA) equipped with 1.7-μm BEH130C18 and 75-μm I.D. × 250-mm reverse-phase column. The analytical column was coupled with Q Exactive Plus high-resolution mass spectrometer (Thermo Fisher Scientific) through an electrospray ionization source. The instrument was operated in a data-dependent mode of acquisition, with a precursor mass spectrometry (MS) scan from m/z of 375–1,600 at *r* = 70,000, followed by the acquisition of 10 MS/MS spectra at *r* = 15,000 by using 26% CID energy setting.

All MS/MS samples were analyzed using Mascot software (version 2.5.1; Matrix Science, London, UK). The Mascot software was calibrated to search SwissProt_2014_01 database (for *S. cerevisiae*, unknown version, 7802 entries, only “F058402”) by assuming the digestion enzyme trypsin and Trembl_2014 database (for fungi, unknown version, 2211048 entries, only “F058400”) also by assuming cleavage by trypsin. The Mascot software was searched using a fragment ion mass tolerance of 0.020 Da and parent ion tolerance of 5.0 PPM. Carbamidomethylation of cysteine was specified as a fixed modification and deamidation of asparagine and glutamine and oxidation of methionine were specified as variable modifications in the Mascot software. Scaffold (version Scaffold_4.4.5; Proteome Software Inc., Portland, OR) was used to validate peptides and proteins identified by performing MS/MS analysis. Peptide identifications were accepted if they could be established at >78.0% probability to achieve an FDR <1.0% by the Scaffold Local FDR algorithm. Protein identifications were accepted if they could be established at >93.0% probability to achieve an FDR <1.0% and contained at least 1 identified peptide. Protein probabilities were assigned using Protein Prophet algorithm (Nesvizhskii et al., 2003). Proteins containing similar peptides and proteins that could not be differentiated based on MS/MS analysis alone were grouped together to satisfy the principles of parsimony.

### *In vivo* Co-immunoprecipitation Assay

*Cryptococcus neoformans* strains HP181 (*pbp1*Δ + *PBP1*::*4xFLAG*) and HPC21 (*pbp1*Δ + *PBP1*::*4xFLAG GFP*::*MKT1*) were grown in liquid YPD medium at 30°C until the OD_600_ reached 0.6–0.8. After incubation, the cells were collected by centrifugation, lysed in lysis buffer by using 10 cycles of the mini-bead beater (90 s homogenization, with 2 min rest), and centrifuged in a microcentrifuge at 13,000 × *g* for 10 min. Supernatant obtained was incubated with the GFP Trap agarose (Chromotek). Immunoprecipitates produced were collected by centrifugation and were washed three times with PBS. Next, the beads were resuspended in Laemmli sample buffer (Bio-Rad), boiled for 10 min, and centrifuged briefly. Supernatant obtained was resolved by SDS-PAGE, and resolved proteins were transferred onto PVDF membranes (Bio-Rad). The membranes were assayed by performing western blotting with anti-GFP antibody (SC-9996; Santa Cruz Biotechnology, Santa Cruz, CA, USA) or anti-FLAG M2 antibody (F3165; Sigma), followed by incubation with horseradish peroxidase-conjugated anti-mouse antibody.

### Sexual Reproduction and Pheromone Gene Expression Assays

Phenotypic analysis of sexual reproduction was conducted as described previously (Park et al., 2016). *MAT*α and *MAT***a** isolates of WT and mutant strains were grown overnight in liquid YPD medium. Next, the cells were washed with water, mixed at equal amounts of cells (~1 × 10^8^ cells), spotted on MS solid medium (Sigma), and incubated in the dark at room temperature for 5–7 days. Images of sexual hyphae were captured using an Eclipse E400 microscope (Nikon) equipped with a DMX1200F camera (Nikon).

Pheromone gene expression was analyzed by culturing *MAT*α and *MAT***a** cells of WT or mutant strains overnight. Next, the cultured cells were mixed at equal density, spotted on V8 solid medium (pH 5), and incubated in the dark at room temperature for 24 h. Samples were collected, frozen quickly, and stored at −80°C. Total RNA was isolated from each sample by using Trizol reagent (Thermo Fisher Scientific), according to the manufacturer's instructions, and complementary DNA was synthesized using an AffinityScript QPCR cDNA Synthesis Kit (Agilent Technologies). Quantitative PCR was performed using the Brilliant III Ultra-Fast SYBR QPCR Mix (Agilent) and a StepOnePlus Real-Time PCR system (ABI). A housekeeping gene *GPD1*, encoding the glyceraldehyde-3-phosphate dehydrogenase (GAPDH), served as a control in the expression analysis.

### *In vivo* Virulence Assay

The strains were cultured in liquid YPD medium at 30°C for 16 h. Next, the cultured cells were collected, washed with sterile PBS, and counted using a hemocytometer, and their final density was adjusted to 1 × 10^7^ CFU/mL. Six- to seven-week-old female BALB/c mice (Daehan BioLink Co., Ltd., Eumseong, Korea) were used for the murine infection model. Groups of six mice were used for survival tests. The mice were anesthetized and infected with 5 × 10^5^ CFU (in 50 μL) of the *C. neoformans* strains through intranasal instillation (Cox et al., 2000). Survival was monitored daily, and moribund mice were sacrificed by CO_2_ administration. Survival curves were generated using the Kaplan–Meier method with Prism 4.0 (GraphPad software), and statistical significance (*p*-values) was assessed using log-rank test.

### Ethics Statement

Protocols for animal care and experiments were conducted in accordance with ethical guidelines established by the Ethics Review Committee for Animal Experimentation (ERCAE) of Handong Global University (HGU). Moreover, all experimental protocols involving vertebrate animals were approved by the ERCAE of HGU (#HGU-20160616-009).

## Results

### Identification of Pbp1-Interacting Proteins

**Figure 1:**
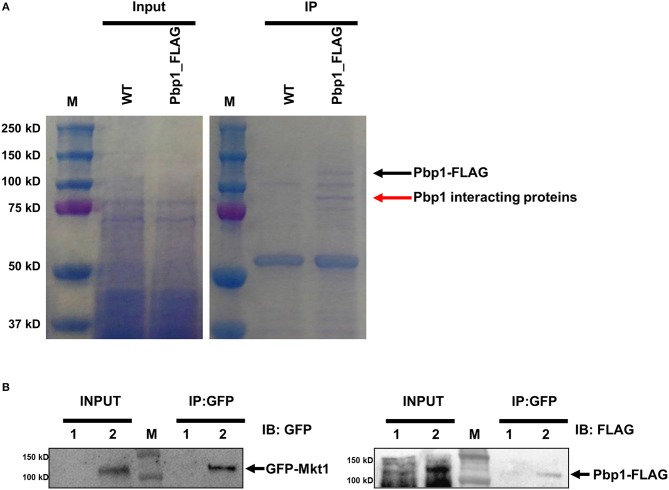
Identification of Pbp1-interacting proteins. **(A)** Coomassie blue-stained gels showing total and Pbp1–FLAG-enriched proteins. M indicates the molecular mass standard. Left gel shows proteins present in soluble lysates before immunoprecipitation. Right gel shows eluted proteins from WT and Pbp1–FLAG-expressing strains. Black arrow indicates the Pbp1–FLAG protein, as determined by performing western blotting. Red arrow indicates a Pbp1-interacting protein excised from the gel for subsequent MS analysis. **(B)** Co-immunoprecipitation assay and western blotting analysis were performed in HP181 (negative control; Pbp1-FLAG, 1) and HPC21 (Pbp1-FLAG and GFP-Mkt1, 2). Co-immunoprecipitation (IP) analysis of *C. neoformans* HP181 and HPC21 strains was performed using GFP-Trap agarose bead. Immunoblotting (IB) analysis was performed subsequently by using anti-FLAG or anti-GFP antibody.

Pbp1 is a putative calcineurin target associated with sexual reproduction and virulence in *C. neoformans*. Pbp1 colocalizes with Cna1, the calcineurin a catalytic subunit, in P-bodies and stress granules in response to heat stress (Park et al., 2016). Therefore, we hypothesized that Pbp1 interacts with Cna1 at 37°C. To test this hypothesis, we used a *C. neoformans* strain expressing 4×FLAG-conjugated Pbp1 (Pbp1-4×FLAG) in a *pbp1*Δ background. FLAG immunoprecipitation were performed from the *pbp1*Δ expressing Pbp1-4×FLAG and the WT *C. neoformans* (H99) strain not expressing Pbp1-4xFLAG (used as a negative control). Recovered proteins were separated in a polyacrylamide gel (Figure 1A). Along with the Pbp1-4xFLAG (black arrow), a protein band was detected (red arrow) in extracts from the Pbp1–FLAG-expressing strain, that was not observed in the Pbp1 capture from WT untagged cell extract and considered a candidate Pbp1 interacting protein. This protein band was excised and further analyzed by MS. Excision of the one band (red arrow) from *in vivo* co-immunoprecipitation analysis and subsequent mass spectrometry resulted in 405 total peptides from 74 proteins in total (Table S1). In all, 18 putative Pbp1-associated proteins were identified (Table 3), with >10% coverage of amino acid sequences. Of these, Mkt1 was identified with 80% sequence coverage in the total Pbp1 protein captured (Table 3).

**Table 3:** Potential Pbp1 associated proteins identified by IP-MS.

	**Locus**	**Protein** ** name**	**Protein function or domain**	**Percentage sequence coverage (%)**	**Number of** ** peptides**
1	CNAG_04808	Mkt1	XPG N-terminal domain-containing protein	80.00	51
2	CNAG_01890	Met6	Cobalamin-independent methionine synthase	68.58	41
3	CNAG_06150	Hsc82	Cytoplasmic chaperone of the Hsp90 family	39.40	21
4	CNAG_00410		RNA recognition motif	29.62	16
5	CNAG_02814	Gut2	Mitochondrial Glycerol-3-phosphate dehydrogenase	23.15	13
6	CNAG_04082	YHR020W	proline-tRNA ligase	24.02	13
7	CNAG_05013	Hrb1	RNP domain-containing protein	29.45	11
8	CNAG_01137	Aco1	Aconitate hydratase	17.90	10
9	CNAG_01726	Spt16	Subunit of the heterodimeric FACT complex	12.75	10
10	CNAG_06387	CDC53	Cullin 1	16.38	9
11	CNAG_01117	HEF3	Translational elongation factor EF-3	11.27	8
12	CNAG_05661	Pob3	Subunit of the heterodimeric FACT complex	18.84	8
13	CNAG_04032	Yta12	AFG3 family protein	14.40	7
14	CNAG_02578	Pch2	Thyroid hormone receptor interactor 13	17.33	7
15	CNAG_03494		Hypothetical protein	17.24	6
16	CNAG_03602	Utp5	U3 small nucleolar RNA-associated protein 5	15.68	6
17	CNAG_05385	Cog6	Uncharacterized protein	10.87	6
18	CNAG_03675	Puf6	Pumilio-homology domain protein	11.20	6

To verify whether Pbp1 interacts with Mkt1, Pbp1–4×FLAG was co-expressed with GFP–Mkt1 and precipitated using GFP-Trap agarose beads. We observed that GFP–Mkt1 co-precipitated with Pbp1–4×FLAG (Figure 1B), suggesting that Mkt1 specifically interacts with Pbp1 in *C. neoformans*.

### Identification and Deletion of *mkt1*

**Figure 2:**
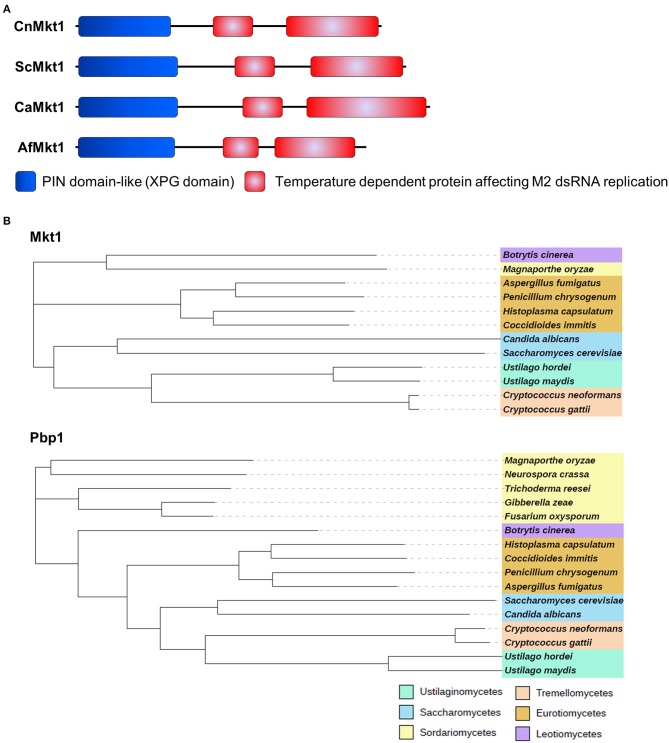
Mkt1 in different fungi. **(A)** Domain architecture of Mkt1 orthologs in different fungi. CnMkt1, *C. neoformans* H99 Mkt1 (XP_012052222); ScMkt1, *S. cerevisiae* Mkt1 S288C (NP_014314); CaMkt1, *C. albicans* SC5314 (XP_720417); AfMkt1, *A. fumigatus* AF293 (XP_753426). **(B)** Phylogenetic tree of Mkt1 and Pbp1 orthologs in different fungal species. Compared sequences were obtained from *Neurospora crassa* OR74A, *Magnaporthe oryzae* 70-15, *Trichoderma reesei* QM6a, *Fusarium oxysporum* Fo47, *Gibberella zeae* PH-1, *Botrytis cinerea* T4, *A. fumigatus* AF293, *Penicillium chrysogenum, Coccidioides immitis* RS, *Histoplasma capsulatum* H88, *C. albicans* SC5314, *C. neoformans* H99, *C. gattii* Ru294, *Ustilago hordei*, and *U. maydis* 521. A phylogenetic tree of Mkt1 orthologs was generated using MEGA 5 software (http://www.megasoftware.net/) with alignment data from ClustalW2. Results obtained using the phylogenetic tree were submitted to iTOL (http://itol.embl.de/) to generate the figure.

Mkt1 (XP_012052222) contains an N-terminal XPG-like domain and is a potential ortholog of *S. cerevisiae* Mkt1 (22% identity). The XPG domain (PIN domain) of Mkt1 is conserved in XPG endonuclease family proteins (Tadauchi et al., 2004). Mkt1 also contains another domain that exerts temperature-dependent effects on the replication of M2 double-stranded RNA (Wickner, 1980, 1987). *S. cerevisiae* and major pathogenic fungi contain Mkt1 orthologs with two conserved domains (Figure 2A). Mkt1 and Pbp1 are widely conserved in most (but not all) ascomycetes and basidiomycetes; however, Mkt1 is not present in fungi belonging to the class *Sordariomycetes* (Figure 2B). To examine Mkt1 function in *C. neoformans*, we generated *mkt1*Δ mutant strains by performing homologous recombination with a dominant drug resistance marker gene. We also generated an *mkt1*Δ + *MKT1* strain in which the *mkt1*Δ mutant was complemented with the wild type *MKT1* gene in *C. neoformans*.

**Figure 3:**
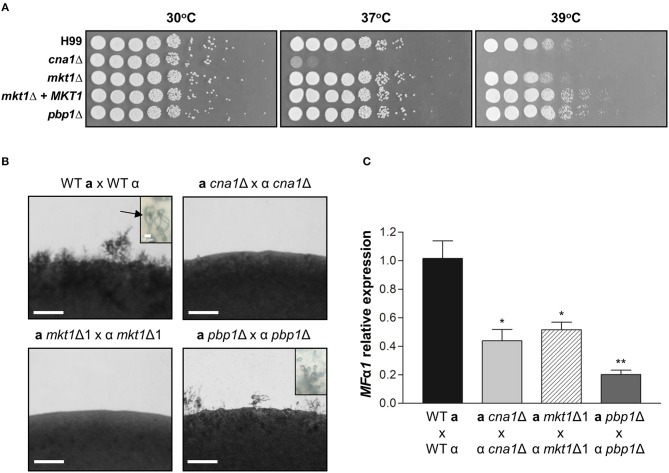
Phenotypes of *mkt1*Δ mutant strains. **(A)** Spot dilution assays with WT (H99), *cna1*Δ (HP242HP242), *mkt1*Δ1 (HPC7), *mkt1*Δ2 (HPC8), *mkt1* + *MKT1* (HPC11), and *pbp1*Δ (HP5HP5) strains. Cells were incubated overnight; diluted by 5-fold; plated on YPD medium; and incubated for 2 days at 30, 37, and 39°C. **(B)** Sexual reproduction assays were conducted for *mkt1*Δ mutant strains. WT (H99α, KN99**a**), α *cna1*Δ (HP242), **a** *cna1*Δ (HP243), α *mkt1*Δ1 (HPC7), **a** *mkt1*Δ (HPC9), α *pbp1*Δ (HP5), and **a** *pbp1*Δ (HP246) strains were co-cultured in the MS medium and were incubated in the dark at room temperature for 7 days. (Scale bars = 200 μm). Images show hyphal growth on the edge of mating patches after incubation. The WT cross between H99α and KN99**a** produced aerial hyphae and basidiospores chain (indicated by arrow). However, the *cna1* and *mkt1* mutant bisexual crosses did not produce hyphae and the *pbp1* mutant bisexual cross had a defect in production of basidiospores. **(C)** The mRNA expression of the pheromone gene (*MF***α***1*) was analyzed in WT, *cna1*Δ, *mkt1*Δ, and *pbp1*Δ strains by performing mating crosses, followed by incubation in the V8 medium at room temperature for 24 h. (H99α × KN99**a** vs. α *cna1*Δ × **a** *cna1*Δ, *p* = 0.0170; H99α × KN99**a** vs. α *mkt1*Δ1 × **a** *mkt1*Δ, *p* = 0.0206; H99α × KN99**a** vs. α *pbp1*Δ × **a** *pbp1*Δ, *p* = 0.0031; α *cna1*Δ × **a** *cna1*Δ vs. α *pbp1*Δ × **a** *pbp1*Δ, *p* = 0.0480; α *mkt1*Δ × **a** *mkt1*Δ vs. α *pbp1*Δ × **a** *pbp1*Δ, *p* = 0.00369) (WT vs. mutant strains, ^*^p ≤ 0.05; ^**^p ≤ 0.01).

Pbp1 is required for proper thermal stress response as the *pbp1*Δ mutant exhibited higher resistance at 39°C when compared to the WT (Park et al., 2016). This raises the possibility that Mkt1 might also be involved in heat stress responses. To examine the role of Mkt1 at high temperature, serially diluted cells were spotted on YPD medium and incubated at different temperatures. The *cna1*Δ mutant strain showed increased heat sensitivity at 37 and 39°C while, as previously observed, the *pbp1*Δ mutant strain was resistant to this thermal stress compared to the WT strain. However, the *mkt1*Δ mutant and WT strains roughly showed the same heat sensitivity, indicating that Mkt1 is not involved in heat stress responses (Figure 3A).

### Mkt1 Is Required for Sexual Reproduction

In *S. cerevisiae*, the Pbp1–Mkt1 complex is required for *HO* endonuclease gene expression (Tadauchi et al., 2004). Because Pbp1 is required for proper sexual reproduction in *C. neoformans*, we examined whether Mkt1 is also required for sexual reproduction in *C. neoformans*. To test the role of the *MKT1* gene in opposite-sex mating, opposite mating type cells of WT and mutant strains were cultured, mixed, and spotted on MS media. In WT crosses, aerial mating hyphae were observed on the edge of mating patches. At tip of hyphae, basidiospores chains were generated. In contrast, the *mkt1*Δ mutant strain showed defective hyphal elongation during sexual reproduction, similar to *cna1*Δ mutant strains (Figure 3B). In α *pbp1*Δ × **a** *pbp1*Δ mutant crosses, aerial hyphae were observed, whereas no basidiospores chains were found. We then examined expression of the pheromone gene *MF*α*1* during sexual reproduction. As shown in Figure 3C, *mkt1* or *pbp1* deletion resulted in decreased expression of *MF*α*1*. These results indicate that both Mkt1 and Pbp1 are required for pheromone gene expression and sexual reproduction in *C. neoformans*.

### Mutation of *mkt1* Attenuates Virulence

**Figure 4:**
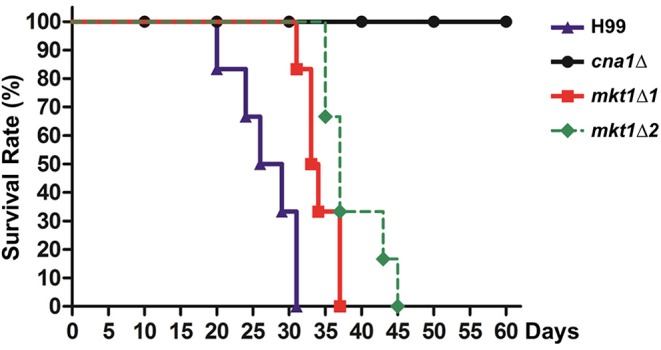
Virulence of the *mkt1*Δ mutant strain in the mouse intranasal inhalation model. WT (H99), *cna1*Δ (HP242HP242), *mkt1*Δ1 (HPC7), and *mkt1*Δ2 (HPC8) strains were grown overnight in YPD liquid medium at 30°C and were washed with PBS. Next, the cells (density, 5 × 10^5^ cells) were inoculated into BALB/c mice through intranasal instillation. Animal survival was monitored for 60 days after the infection. [H99 vs. *mkt1*Δ1 (HPC7), *p* = 0.0023; H99 vs. *mkt1*Δ2 (HPC8), *p* = 0.0008; H99 vs. *cna1*Δ (HP242), *p* < 0.0001; *cna1*Δ (HP242) vs. *mkt1*Δ1 (HPC7), *p* < 0.0001; *cna1*Δ (HP242) vs. *mkt1*Δ2 (HPC8), *p* < 0.0001].

Because the *pbp1*Δ mutant strain showed attenuated virulence in a murine intranasal inhalation model, we hypothesized that Mkt1 is also required for the virulence of *C. neoformans* in this infection model. To test this, animals were infected with WT, *cna1*Δ, and two independent *mkt1*Δ mutant strains. The *mkt1*Δ mutant strain (median survival, 34 and 37 days) showed attenuated virulence compared with the WT strain (median survival, 28 days) (Figure 4). These results support a role of Mkt1 in fungal virulence in the mouse intranasal inhalation model and suggest that Mkt1 is required for the full virulence of *C. neoformans*.

## Discussion

Pbp1 is a Pab1-interacting protein that regulates polyadenylation by regulating Pab1 in *S. cerevisiae* (Mangus et al., 1998). Pbp1 interacts with other proteins such as Lsm12, Pbp4, and Mkt1 and localizes to and promotes the formation of stress granules and P-bodies (Mangus et al., 2004b; Swisher and Parker, 2010). In *C. neoformans*, Pbp1 also colocalizes with the P-body component protein Dcp1, stress granule component protein Pub1, and calcineurin catalytic subunit Cna1 (Park et al., 2016). In the present study, we hypothesized that Pbp1 interacts with the calcineurin complex and additional P-body, or stress granule components. In total 405 peptides representing 74 total proteins from a protein gel band were detected (a red arrow) in the Pbp1–FLAG-expressing strain (Table S1). Of these proteins, Mkt1 was found to be a Pbp1-interacting protein. Other proteins in the band were RNA-binding proteins (CNAG_00410, Hrb1, Puf6, and Pab1), heterodimeric FACT complex subunits (Spt16 and Pob3), and translation initiation/elongation factors. In both *S. cerevisiae* and *T. brucei*, Pbp1 interacts with poly(A)-binding proteins Pab1 and Lsm12 (Mangus et al., 1998; Singh et al., 2014). The present study identified four Pab1 peptides in the band, suggesting Pbp1 could interact with Pab1 in *C. neoformans*. However, we could not demonstrate whether Pbp1 interacts with Cna1 in *C. neoformans*. We speculate that this could be due to assay sensitivity limitation to detect all Pbp1 interacting proteins. This result does not rule out the possibility that Mkt1 is a calcineurin substrate, it is well-known that most protein phosphatases interact weakly and transiently with their substrates and do not form detectable complexes.

Mkt1 contains an N-terminal PIN domain that belongs to a large nuclease superfamily. However, the N-terminal PIN domain of Mkt1 lacks several amino acids necessary for cofactor- and DNA-binding activity, suggesting that it may not possess nuclease activity. Although the biochemical function of Mkt1 has not been examined in detail, Mkt1 is necessary for post-transcriptional gene regulation (Tadauchi et al., 2004; Devaux et al., 2010; Singh et al., 2014). Importantly, Mkt1 interacts with Pbp1 and other RNA-binding proteins to form an Mkt1-mediated regulator complex that maintains mRNA stability (Singh et al., 2014). Our study suggests that Mkt1 might also form complexes with other RNA-binding proteins such as Hrb1 and Puf6 in *C. neoformans* (Table 3), raising the possibility that these novel complexes may be involved in mRNA regulation.

As Mkt1 forms the Mkt1-Pbp1 complex, we hypothesized that Mkt1 and Pbp1 perform a similar role in *C. neoformans*. We examined Mkt1 function in *C. neoformans* and found that *mkt1* deletion conferred defects in virulence (Figure 4) similar to *pbp1* deletion, suggesting that Mkt1 plays a similar role as Pbp1 in virulence. However, the *mkt1* deletion mutant strain showed nearly a wild-type phenotype at high temperature (39°C; Figure 3A). Thus, our results suggest that Mkt1 plays a different role from Pbp1 in stress responses to high temperature. During bisexual reproduction, aerial hyphae and basidiospores were not observed in α *mkt1*Δ × **a** *mkt1*Δ mutant crosses (Figure 3B). However, α *pbp1*Δ × **a** *pbp1*Δ mutant crosses showed decreased filamentation and no basidiospore chain formation. Based on these data, we speculate that both Mkt1 and Pbp1 are required for proper sexual reproduction, but the roles of Mkt1 and Pbp1 during mating processes are slightly different. Further epistatic and biochemical analyses of the roles of the Mkt1–Pbp1 complex will contribute to determine molecular mechanisms that regulate sexual development and virulence in fungi.

## Conclusion

In this study, we identified the Mkt1 as a Pbp1 interacting protein. In addition, we demonstrated that Mkt1 plays an important role in sexual development and virulence in *C. neoformans*. Our finding provides further understanding of sexual development and virulence mechanism of *C. neoformans*.

## Data Availability Statement

All datasets generated for this study are included in the manuscript/Supplementary Files.

## Ethics Statement

The animal study was conducted in accordance with ethical guidelines established by the Ethics Review Committee for Animal Experimentation (ERCAE) of Handong Global University (HGU). Moreover, all experimental protocols involving vertebrate animals were approved by the ERCAE of HGU (#HGU-20160616-009).

## Author Contributions

H-SP designed the experiments. Y-ES, W-HJ, CF, and S-HO performed the experiments. MC, JH, J-HK, and H-SP analyzed the data and wrote the manuscript.

### Conflict of Interest

The authors declare that the research was conducted in the absence of any commercial or financial relationships that could be construed as a potential conflict of interest.

## Supplementary Material

Table S1 List of potential Pbp1 associated proteins and peptides identified by IP-MS *C. neoformans*.
